# Multicentric reticulohistiocytosis with extra-mammillary Paget’s disease: a case report

**DOI:** 10.1186/2047-783X-18-38

**Published:** 2013-10-29

**Authors:** Xinyan Huang, Limei Zhang, Songzhao Zhang

**Affiliations:** 1 Department of Dermatology, The Second Affiliated Hospital of Zhejiang University School of Medicine, Hangzhou 310009, China; 2 Department of Dermatology, The Third Affiliated Hospital of Zhejiang Chinese Medical University, Hangzhou 310000, China; 3 The Department of Clinical Laboratory Medicine, The Second Affiliated Hospital of Zhejiang University School of Medicine, Hangzhou 310009, China

**Keywords:** Multicentric reticulohistiocytosis (MRH), Extra-mammillary Paget’s disease (EMPD), First report

## Abstract

Multicentric reticulohistiocytosis (MRH) is a very rare systemic disease with variable phenotypic presentation and a high rate of misdiagnosis. Here we describe a patient with MRH and extra-mammillary Paget’s disease (EMPD), a diagnosis that has not previously been described in the literature.

## Background

Multicentric reticulohistiocytosis (MRH) is a very rare disease and was first identified in 1936 [1-6]. The disease can involve the skin, mucosa, joints and/or muscles. Owing to the frequency of accompanying joint symptoms, MRH is easily misdiagnosed as rheumatoid arthritis or dermatomyositis. Patients can also present with co-morbid tumors. Here, we describe a case with co-morbidity of MRH and extra-mammillary Paget’s disease (EMPD).

## Case presentation

On 10 May 2012, a 47-year-old woman was admitted to our department because of a 1-year history of polyarthralgia and multiple papules that had first appeared 2 months prior. Five years previously, the patient had noticed an edematous rash on her left vulva; the rash was accompanied by a persistent itch. Scratching the rash resulted in the release of clear fluid. She went to local hospitals several times and was diagnosed with eczema. One year prior to presentation at our clinic, the patient began to experience symmetrical polyarthralgia, which was typically mild but exacerbated with exercise. The patient’s pain radiated from her ankle to her knee, and also from her finger to her palm, wrist, elbow, shoulder and spine, in series. The patient eventually experienced difficulty with flexion of both upper and lower limbs, such that walking became difficult. Two months later, she noticed that multiple red papules had appeared on her neck, upper chest and the distal portion of her dorsal finger. At each of several local hospitals, the patient was diagnosed with rheumatoid arthritis and prescribed oral medication such as ibuprofen tablets; none of these had any effect. Six months ago, the patient lost 3 kg from her baseline weight. She was married with a son and a daughter, both of whom were healthy. Her father and younger brother had died of liver cancer. Notably, the patient had been menopausal for 3 years. Her medical history and personal history were normal.

**Figure 1:**
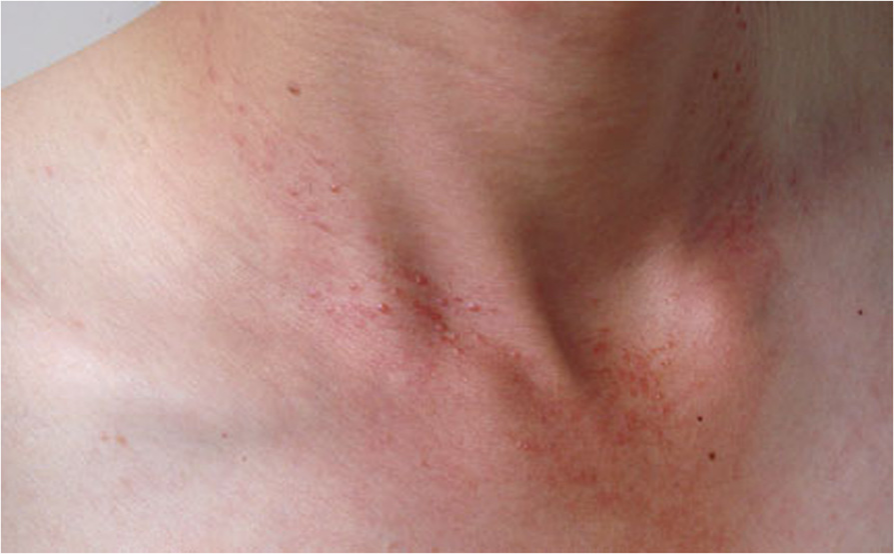
Picture of neck.

**Figure 2:**
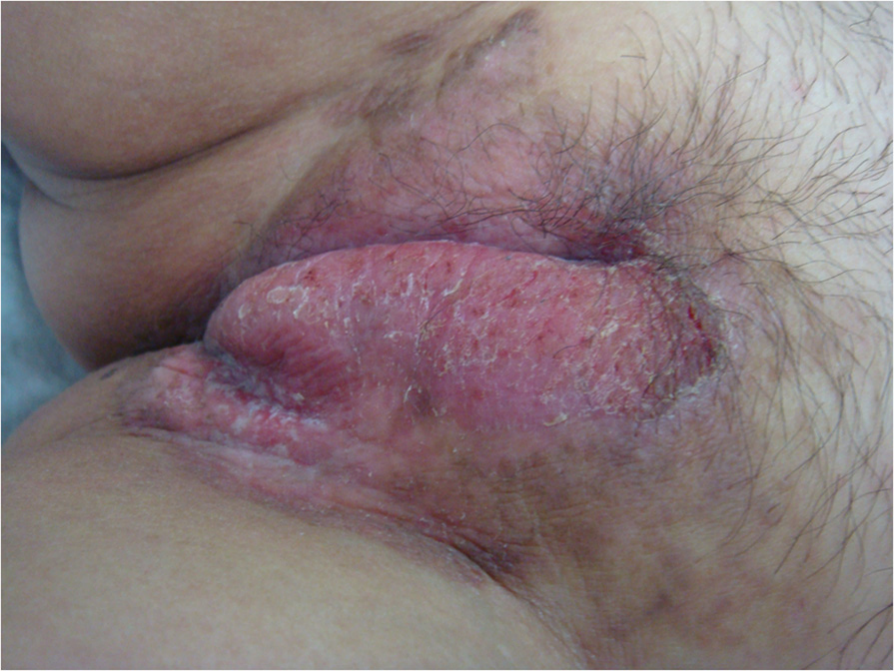
Picture of vulva.

**Figure 3:**
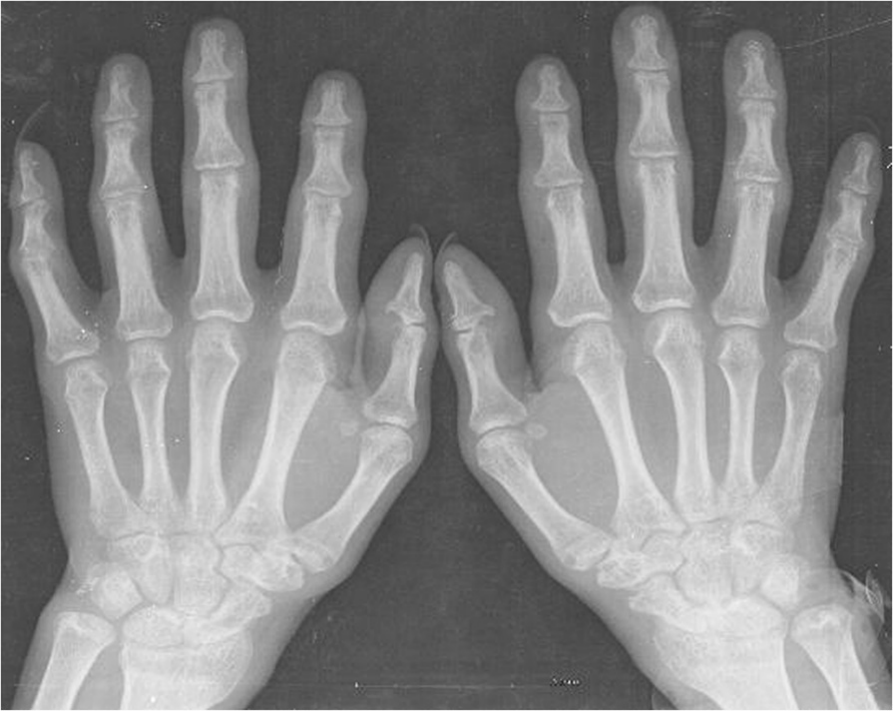
**X-ray examination.** This shows osteoporosis at both ends of the two hands, irregular bone destruction on part of the proximal interphalangeal joint, narrowing of local joint spaces, small fibrocystic change on part of the carpal bones and the first metacarpal base of both hands and light soft-tissue swelling.

Physical examination revealed multiple light-red papules on the forehead, ears, scalp, neck (Figure 1), upper chest and distal dorsal finger. The papules were about 0.5 mm in diameter, with variable distribution. The surface of the papules was lighter than normal skin. Despite the absence of limb swelling, the patient experienced difficulty in moving her upper-limb joints, knees and spine. The red papules on the left labium majus were clear and raised, with central ulcers and white scales on the surface (Figure 2). The patient’s inner thighs were covered by dark brown rashes. An X-ray examination (Figure 3) revealed osteoporosis in both hands, irregular bone destruction in the proximal interphalangeal joint, narrowing of local joint spaces, small fibrocystic changes in the carpal bones and the first metacarpal base of each hand and minimal soft-tissue swelling. The knees appeared to be normal.

**Figure 4:**
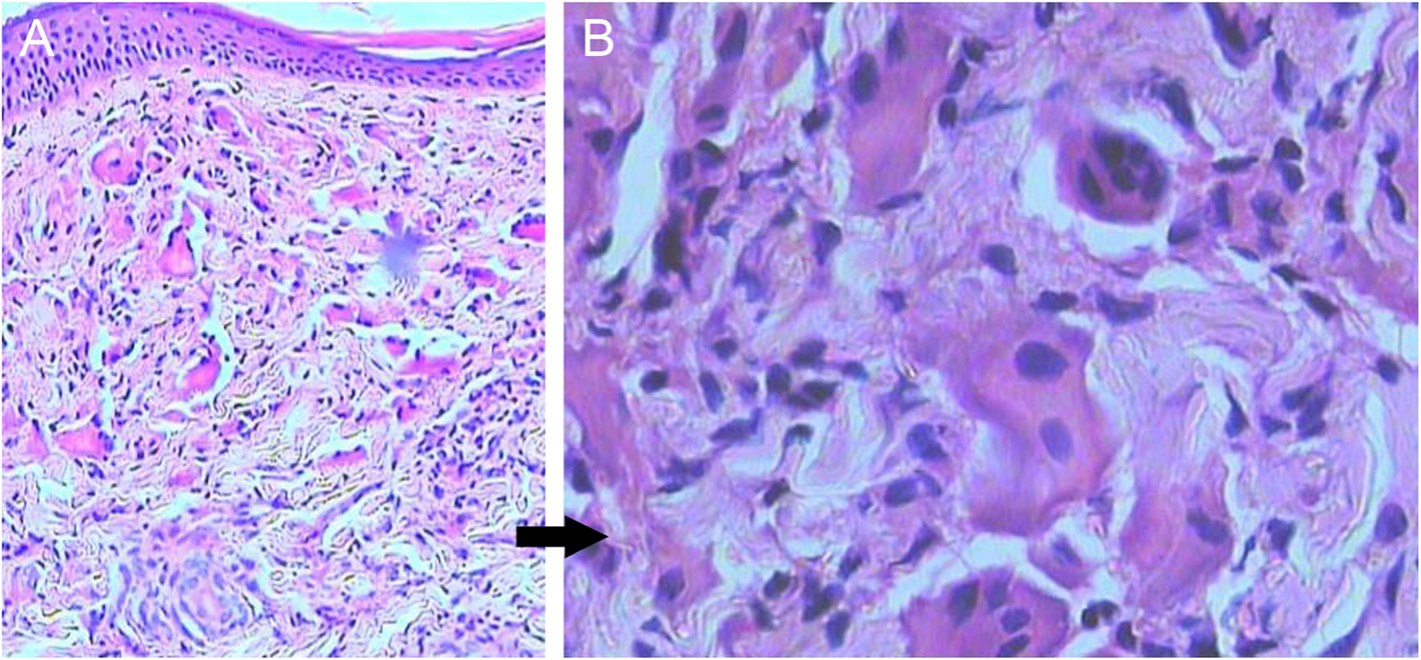
**Pathology of the lesion on the left-posterior side of the neck.** Light atrophy of the epidermis and an accumulation of nodular tissue cells on the shallow dermis are visible. Multinuclear giant cells and tissue cells are scattered among the dermal collagen bundles and there is a little lymphocytic infiltration of the shallow vessels (see Figure 3). **(A)** Magnification 200×, **(B)** magnification 400 ×.

**Figure 5:**
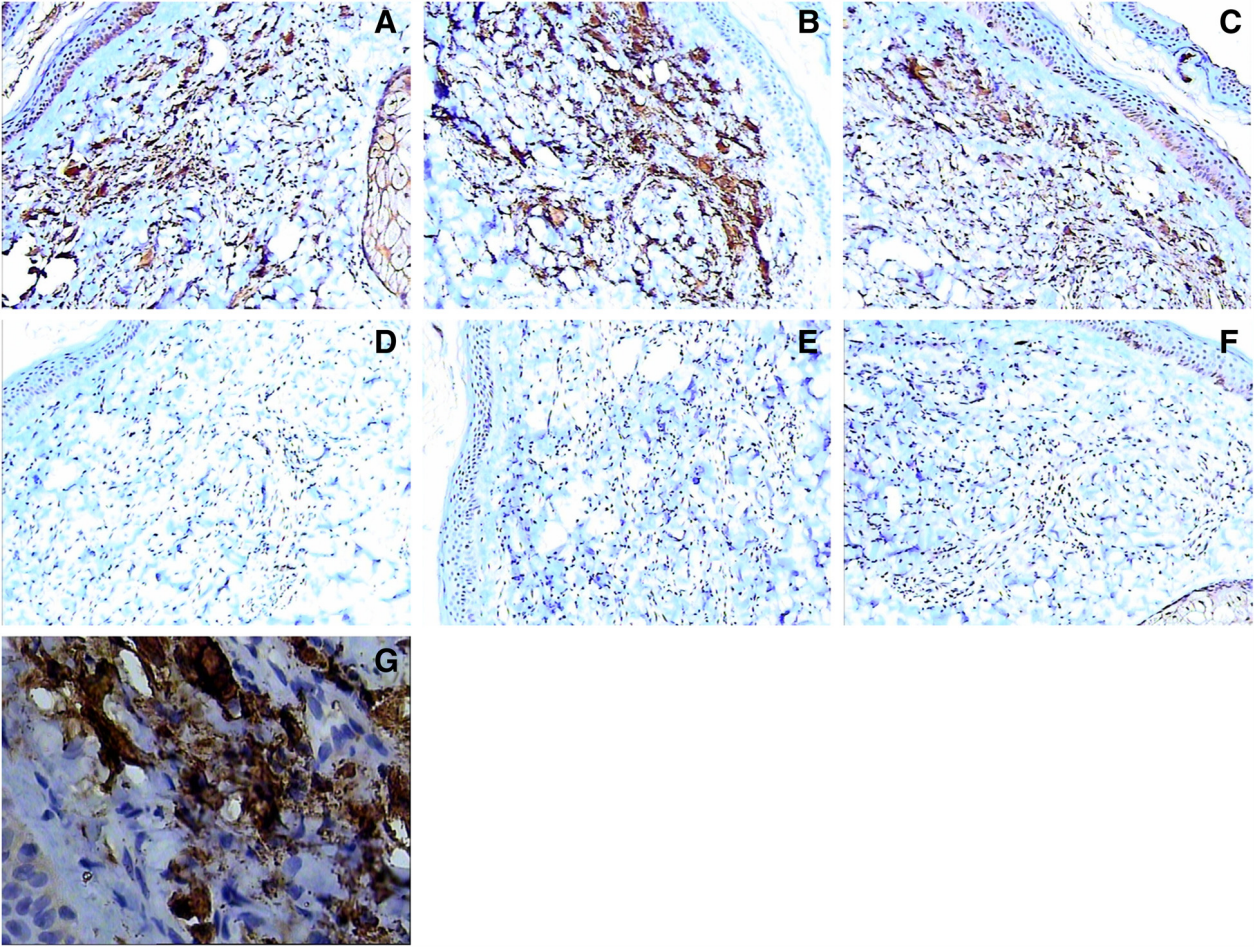
**Immunohistochemical examination. (A)** and **(B)** lysozyme ++. **(C)** EMA -. **(D)** CD1a -. **(E)** S-100 -. **(F)** CD68 ++. **(G)** AAT ++. (Magnification 200×).

**Figure 6:**
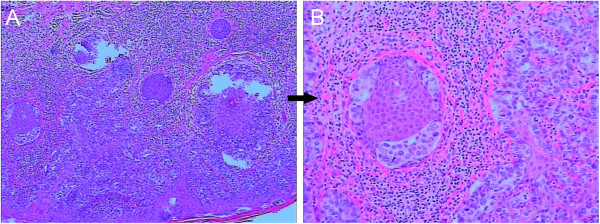
**Skin biopsy of the cunnus.** There are round or ovoid Paget cells where the cell body is quite big and the nucleus is big, trachychromatic and sometimes pushed to one side of the cell. There is some karyokinesis with single or areatus cells and adenoid differentiation. **(A)** Magnification 200×, **(B)** magnification 400 ×.

The following auxiliary examinations were performed as well: a blood test, urine test, stool analysis, blood biochemistry, serum levels of T3/T4, serum immunoglobulin and complement, anti-CCP (-), blood ANA (-) and blood RF (-). The results of all tests were normal. The results of the chest X-ray and electrocardiogram were also normal. Ultrasound examination revealed a 4 × 2.3-cm intrauterine hypoechoic mass, bordering the muscle, which appeared to contain high levels of calcium. Notably, the lymph nodes were found on both sides of the neck, the armpit, and the groin. The liver, spleen, bladder and pancreas were normal. Pathological analysis of the neck skin (Figure 4) revealed light atrophy of the epidermis, the accumulation of nodular tissue cells in the shallow dermis, scattered multinuclear giant cells and tissue cells among the dermal collagen bundles and minimal lymphocytic infiltration in the superficial vessels. Granulomatous proliferation and frosted glass-like changes can be distinguished from sarcoidosis, which presents with an epithelioid cell granuloma with clear boundaries. Immunohistochemical analysis of a skin biopsy from the neck (Figure 5) revealed lysozyme ++, CD68 ++, AAT ++, CD1a -, S-100 - and EMA -. A skin biopsy of the cunnus revealed epidermal acanthosis and Paget cell polymegathism (Figure 6). The Paget cells appeared to have abundant cytoplasm and were either pale in color or transparent. The cells were free of intercellular bridges but exhibited large and trachychromatic nuclei, which were sometimes pushed to one side of the cell. The cells sometimes displayed a karyokinetic aspect, leading to adenoid differentiation of single cells. Further immunohistochemical examinations: CK7 ++, CK18 ++, CK19 ++, CK20 –, CEA part +, CA125 –, ER –, PR –, P53 + and CDX-2 –.

The clinical manifestation, auxiliary examinations and pathological examination suggested a diagnosis of concomitant MRH and EMPD. A regimen of tripterygium glycosides (a traditional Chinese medicine, which is immunosuppressant, 30 mg/pc) 6 pcs/d was recommended, to avoid any adverse effects. Routine blood examination was performed to monitor the patient’s hepatic and renal function. The patient was also treated with prednisone 5 mg/pc (every week for 1 month, and then once a month), acetate tablets (20 mg/d) and surgical intervention when necessary.

Two weeks later, the patient reported that her arthralgia had improved noticeably, which led to a reduction in the number of papules as well as an improved quality of life. The patient was transferred to the gynecology department and the genital tumor was extensively resected. No infiltration of nearby tissues and organs was found. Colonoscopy and colposcopy results were unremarkable. A telephone follow-up at 1 month revealed that the patient’s MRH was under control, and the patient was engaged in ongoing treatment for her EMPD.

### Discussion

The pathogenesis underlying MRH remains to be determined. The condition is more than twice as common in women as men, and typically affects middle-aged adults [1]. This condition is often co-morbid with tumors, diabetes mellitus, hyperlipidemia and hypothyroidism [7,8]. In the patient presented here, the clinical manifestations included a rash and painless papulonodules. In MRH patients, 60% report concomitant arthritis of the peripheral joints and 50% report issues related to mucus membranes or muscles [9]. A definitive diagnosis requires a skin biopsy [10,11]. There is no specific treatment for this disease, but inflammation is kept under control through the use of glucocorticoids, immunosuppressants, biological agents, TNF-α, IL-1β, tripterygium glycosides and NSAIDs.

EMPD is considered to be an eczematoid carcinoma of the breast, which is an adenocarcinoma that is rarely observed on the skin. EMPD typically affects the vulva. Patients are advised to undergo surgery owing to the risk of relapse.

This patient presented symptoms of vulvar malignancy prior to MRH. Her MRH was originally misdiagnosed as rheumatoid arthritis and her EMPD of the vulva was initially misdiagnosed as eczema. Treatments based on these diagnoses were futile.

We have not been able to find any obvious similarities in the pathophysiology of MRH and EMPD. Some think that MRH is a real paraneoplastic syndrome [12] and related to malignancy. However, the underlying relation is still not determined [13] even though there have been several reports of the co-morbidity of MRH and urologic neoplasms. Further studies are needed to investigate the relation between MRH and EMPD for this case.

## Conclusions

MRH is a rare disease that is very similar to rheumatoid arthritis and diabetes mellitus, which often leads to misdiagnosis. Co-morbidity with EMPD can make an accurate diagnosis even more challenging [2-6]. This case report should aid medical practitioners if they encounter similar clinical presentations.

## Consent

Written informed consent was obtained from the patient for publication of this case report and any accompanying images. A copy of the written consent is available for review by the Editor-in-Chief of this journal.

## Abbreviations

EMPD: Extra-mammillary Paget’s disease; IL: Interleukin; MRH: Multicentric reticulohistiocytosis; NSAIDs: Non-steroidal anti-inflammatory drugs; TNF: Tumor necrosis factor.

## Competing interests

The authors declare that they have no competing interests.

## Authors’ contributions

XYH conceived and designed the experiments and wrote the manuscript. LMZ conceived and designed the experiments. SZZ performed the operation. All authors read and approved the manuscript.
